# Activation of GPR35 in the Anterior Cingulate Cortex Alleviates Neuropathic Pain and Depression‐Related Behavior

**DOI:** 10.1002/cns.70852

**Published:** 2026-03-31

**Authors:** Jianling Xu, Jingyong Zhou, Xiaojun Li, Tingting Qu, Changjian Zheng, Qingyu Cheng, Xiuyang Lei, Weidong Yao, Yongquan Chen, Bin Wang

**Affiliations:** ^1^ Department of Anesthesiology The First Affiliated Hospital of Wannan Medical College, Yijishan Hospital Wuhu China; ^2^ Department of Pain Medicine The First Affiliated Hospital of Wannan Medical College, Yijishan Hospital Wuhu China; ^3^ Department of Neurology The First Affiliated Hospital of Anhui Medical University Hefei China; ^4^ Anhui Province Key Laboratory of Non‐Coding RNA Basic and Clinical Transformation Wuhu China

**Keywords:** anterior cingulate cortex, G protein‐coupled receptor 35, L‐Kyna, neuropathic pain, Nr4a1

## Abstract

**Background:**

Neuropathic pain (NP) is frequently accompanied by anxiety and depression, and current treatments do not adequately address this comorbidity. The anterior cingulate cortex (ACC) plays a central role in sensory and emotional processing. However, the molecular pathways that connect these functions remain unclear. G protein‐coupled receptor 35 (GPR35), an orphan receptor enriched in neurons, has been implicated in neuroinflammation and pain signaling. However, its specific involvement in NP and associated affective disturbances has not been fully elucidated.

**Methods:**

Peripheral blood GPR35 expression was measured in human patients with NP and healthy controls. In mice, chronic constriction injury (CCI) was used to induce NP. Lentiviral knockdown or overexpression of GPR35 was performed in ACC cells. Behavioral assays were used to assess mechanical and thermal sensitivity, locomotor and anxiety metrics, cognitive performance, and depression‐related behaviors. Molecular analyses included western blotting, RT‐qPCR, immunofluorescence, RNA sequencing, and co‐immunoprecipitation. Additional Nr4a1 knockdown and L‐kynurenine (L‐Kyna, a GPR35 agonist) administration were used to validate pathway involvement.

**Results:**

Patients with NP had higher circulating GPR35 levels, which positively correlated with pain intensity. CCI induced a time‐dependent increase in GPR35 expression in the ACC of mice, accompanied by hypersensitivity and emotional disturbances. GPR35 knockdown in the ACC worsens mechanical and thermal hypersensitivity, impairs cognition, increases depression‐related behaviors, and amplifies microglial activation and pro‐inflammatory cytokine production. GPR35 overexpression reversed these effects by reducing hypersensitivity, improving affective behaviors, and restoring the inflammatory balance. Transcriptomic and biochemical analyses identified Nr4a1 as a key downstream effector of GPR35, and Nr4a1 knockdown eliminated the protective effects of GPR35 overexpression. GPR35 primarily regulated the PI3K/AKT pathway. Treatment with L‐Kyna reduced pain hypersensitivity, improved depression‐related behaviors, and decreased neuroinflammation in CCI mice.

**Conclusions:**

GPR35 is an essential regulator of NP and pain‐related affective disturbances in the ACC. Its effects are mediated through the Nr4a1‐dependent activation of the PI3K/AKT pathway and suppression of neuroinflammation. The pharmacological activation of GPR35 using L‐Kyna provides analgesic and antidepressant benefits, highlighting GPR35 as a promising therapeutic target for NP and its emotional comorbidities.

## Graphical abstract

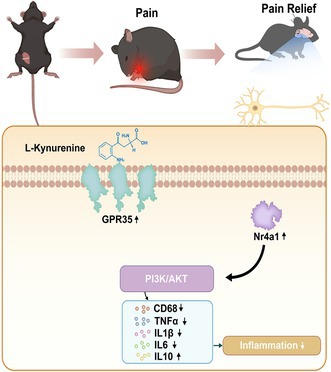

## 1 Introduction

Neuropathic pain (NP) is a prevalent and incapacitating chronic pain disorder that arises from lesions or pathologies affecting the somatosensory nervous system. This condition is frequently comorbid with psychiatric manifestations, including anxiety and depression, and conventional therapeutic interventions demonstrate limited efficacy [1, 2, 3]. Notably, psychopathological alterations may exacerbate pain chronicity and amplify pain perception, establishing a reciprocal reinforcement between nociceptive processing and emotional dysregulation [4, 5]. Consequently, the development of novel therapeutic strategies targeting both NP and their associated psychiatric comorbidities is a critical and unmet need in clinical neuroscience.

Peripheral nerve injury triggers neuroinflammation, subsequently resulting in the development of NP [6, 7]. The anterior cingulate cortex (ACC) plays a critical role in mediating emotional processing and sensory perception [8, 9, 10]. Following neural injury, neuronal activation occurs within the ACC, and suppression of central plasticity in this region exerts analgesic, anxiolytic, and antidepressant effects [1, 11, 12, 13]. Furthermore, peripheral nerve injury induces elevated levels of inflammatory factors in the contralateral ACC region, which are implicated in the persistence of pain and associated negative emotional states [14, 15]. However, the precise molecular pathways through which the ACC modulates sensory perception and emotional responses are yet to be fully elucidated.

G protein‐coupled receptors (GPCRs), which belong to the seven‐transmembrane‐domain protein superfamily, serve as key mediators of intracellular signaling and actively participate in diverse physiological and pathological processes, including nociceptive transmission [16, 17, 18]. Nervous‐system GPCRs have enhanced our understanding of NP pathophysiology and present promising therapeutic targets [19]. GPR35, an understudied orphan receptor [20], is expressed in neurons, glia, macrophages, and monocytes. Emerging evidence implicates its functional significance in neuroinflammatory cascades, pain signaling, and NP development [21, 22] with the development of specific agonists/antagonists for this receptor becoming a focus in drug research [23]. Clinical sample analysis demonstrated significant upregulation of GPR35 expression in whole blood from patients with NP. Furthermore, there was a significant positive correlation between GPR35 expression and subjective pain intensity scores assessed using the McGill Pain Questionnaire. In the chronic constriction injury (CCI) mouse model, we observed dynamic changes in ACC GPR35 expression: upregulated from day 3 post‐surgery and peaking at day 7. This spatiotemporally specific expression pattern indicates GPR35's potential role in NP chronicity. Preliminary data also suggest the potential involvement of GPR35 in the cognitive impairment and affective disturbances associated with Alzheimer's disease pathology [24]. Recently, GPR35 has gained substantial attention as a novel molecular target for NP treatment. Although substantial progress has been made in understanding ligand recognition and signal transduction mechanisms, the mechanisms of GPR35 in NP remain unclear. Specifically, the precise involvement of GPR35 in modulating NP phenotypes and depression‐related behaviors in murine models, along with its underlying molecular mechanisms, are yet to be elucidated.

In this study, we aimed to evaluate the specific role of GPR35 expression in the ACC region with NP. We evaluated GPR35 expression in human patients with NP. Additionally, CCI mouse models were used to validate the NP‐related functional and molecular biological changes under physiological and pathological conditions.

## 2 Materials and Methods

### 2.1 Participants and Animals

This study strictly followed the guidelines of the International Association for the Study of Pain [25] for reporting in vivo experiments. The study has been registered in the “Chinese Clinical Trial Registry” (Registration Number: ChiCTR2500113757). The experimental protocol was approved by the Medical Ethics Committee of Wannan Medical College (Approval Number: 2023‐176). Each participant signed an informed consent form.

All animal experimental protocols were reviewed and approved by the Experimental Animal Welfare and Ethics Committee of Wannan Medical College (Approval Number: WNMC‐AWE‐2023417).

Patients with NP, diagnosed with sciatic nerve compression caused by lumbar disc herniation, were selected from the Department of Spinal Surgery of the First Affiliated Hospital of Wannan Medical College. Exclusion criteria: (1) having chronic diseases such as hypertension and diabetes; (2) having undergone surgery for lumbar disc herniation; (3) unable to cooperate during blood draw; (4) ASA grade > III; (5) Participants and their family members refuse to participate in this project. The healthy control group (Control) was recruited from individuals undergoing routine physical examination. Peripheral blood was drawn from the participants, and expression levels of GPR35 in whole blood from the two groups of participants were analyzed. All participants completed the McGill Pain Questionnaire assessment [26].

Adult male C57BL/6J mice (8 weeks old, 20–30 g) were maintained under specific‐pathogen‐free conditions with standardized environmental parameters, five mice in a numbered cage, ambient temperature maintained at 21°C–23°C, and 12 h light/dark cycles.

### 2.2 CCI

The CCI model was established under isoflurane‐induced anesthesia (induction: 4%, maintenance: 2%–2.5% in 100% O_2_). The left sciatic nerve and its major branches (the sural, common peroneal, and tibial nerves) were carefully exposed using a muscle‐splitting approach. Three microsurgical ligatures (4–0 chromic gut sutures) were loosely placed around the nerve trunk with approximately 1 mm spacing, ensuring minimal compression while restricting nerve mobility. The sham‐operated group underwent an identical surgical exposure without nerve ligation. Subsequently, the skin was closed with a 6–0 suture.

### 2.3 Behavioral Test

The experimental cohorts were allocated using computer‐generated randomization sequences. Animals underwent progressive habituation to the testing environment, consisting of 3 days of facility acclimation followed by ≥ 60 min of chamber‐specific adaptation prior to each behavioral session. All experimental procedures were performed by operators blinded to the group allocation, with the test order counterbalanced across experimental conditions.

#### 2.3.1 Quantitative Mechanosensitivity Assessment

Following a 30 min habituation period on an elevated stainless steel mesh platform (100 × 50 cm), mechanical allodynia was evaluated using calibrated Aesthesio von Frey filaments (0.008–2.0 g; Danmic Global, San Jose, CA, USA). Stimuli were delivered through mesh apertures via perpendicular application to the mid‐plantar surface until 90° filament deflection was achieved, maintaining a consistent 3 s stimulus duration. Non‐responsive trials triggered the sequential application of ascending filament forces according to standardized protocols. The 50% paw withdrawal threshold was calculated using Dixon's up‐down paradigm [27], with inter‐stimulus intervals ≥ 5 min to prevent sensitization. The mechanical withdrawal threshold was determined using an ascending stimulus protocol initiated with a 0.008‐g filament. Each calibrated filament was applied perpendicularly to the plantar surface with consistent force and duration (3 s per application) for 10 trials per filament. Paw withdrawal responses were quantitatively recorded and the mechanical threshold was operationally defined as the minimal force required to elicit a 50% response frequency across consecutive trials [1]. The stimulus application followed a standardized spatial and temporal pattern to ensure test reliability.

#### 2.3.2 Thermal Nociception Assessment

Thermal hyperalgesia was assessed using a Hargreaves apparatus (Plantar Test 37370; Ugo Basile, Gemonio, Italy). Mice were individually placed in transparent polycarbonate chambers (4.5 × 3.0 × 10 cm) positioned on a temperature‐controlled glass surface, allowing a 30 min acclimatization period. A focused radiant heat beam was applied to the mid‐plantar area of the hind paw, maintaining a minimum 3 min interval between stimuli to avoid thermal sensitization. The system automatically synchronized the activation of the heat source and digital timer that ceased upon the detection of paw withdrawal. Positive responses were characterized by the swift retraction of the paw from the heat stimulus. Each animal underwent three consecutive trials, with the average withdrawal latency recorded as the thermal nociceptive threshold, in accordance with standardized methodologies [28]. A single heat stimulus was no longer than 10 s, and the total daily stimulus was no more than five times; if the animal appeared to lick its paws, limp, or have skin lesions, the experiment was immediately stopped.

#### 2.3.3 Open Field Test (OFT)

The testing chamber was operated under standardized environmental conditions with controlled illumination, temperature, and humidity. A square arena (40 × 40 × 40 cm) constructed of opaque acrylic was used as the testing apparatus. Mice were individually placed in the center of an open field and allowed to explore for 5 min. Behavioral endpoints included the central quadrant occupancy time (expressed as a percentage of the total session duration), frequency of central zone entries, ambulatory activity (total locomotion distance), and spatial navigation patterns tracked using video recording systems. Data acquisition followed established ethological assessment protocols, with automated quantification of locomotor and anxiety‐related behaviors.

#### 2.3.4 Novel Object Recognition (NOR)

The same experimental apparatus used in the open‐field arena for the OFT was equipped with an overhead‐mounted camera for behavioral tracking and analysis. The protocol comprised two distinct phases: habituation and evaluation. During the habituation phase, two geometrically identical objects were diagonally positioned in opposite corners of the arena, maintaining a standardized distance of 5 cm from the adjacent walls. Mice were introduced to the arena facing the central area to ensure an equidistant orientation relative to both objects. Object exploration was quantified over a 10 min interval, with exploration defined as direct nasal or oral contact with the object or proximity within 1.0 cm. Exploratory behaviors included active sniffing or forepaw contact. Static climbing without investigative movements was excluded from the analysis. The evaluation phase, conducted 24 h post‐habituation, involved replacing one cylindrical object with a novel cubic object of distinct coloration. The exploration time for both novel and familiar objects was recorded during a 5 min test session, and behavioral parameters were analyzed using the ANY‐maze software version 6.0.

#### 2.3.5 Tail Suspension Test (TST)

The TST was used to evaluate depression‐related behavioral manifestations. The distal 1.0 cm of each mouse tail was secured using a laboratory‐grade adhesive tape and suspended in a calibrated mounting apparatus within the observation chamber. The experimental sessions consisted of a 6 min observation period, with the inactivity duration (defined as the absence of limb or body movements) during the terminal 5 min interval quantified as the primary outcome measure. Behavioral parameters were captured using digital recording systems and analyzed using automated behavioral analysis software to ensure a standardized assessment of immobility time.

#### 2.3.6 Forced Swimming Test (FST)

The FST was used to assess depression‐like behavioral phenotypes in mice. Individual animals were placed in a transparent cylindrical vessel containing warm water, to a depth of 15 cm. Following a 6 min test duration, the immobility time during the terminal 5 min interval was quantified as the primary behavioral metric. Immobility was operationally defined as passive floating behavior characterized by minimal limb movement while maintaining buoyancy and respiratory function. Behavioral parameters were captured and analyzed using an automated video tracking system with integrated behavioral recognition algorithms to ensure the objective quantification of immobility duration.

#### 2.3.7 Sucrose Preference Test (SPT)

The SPT was preceded by a brief isolation period during which mice were individually housed with ad libitum access to dual 1% (*w*/*v*) sucrose reservoirs for 24 h of habituation. Following this acclimatization phase, one sucrose reservoir was replaced with distilled water for an equivalent duration. Subsequent implementation of a 24 h caloric‐ and fluid‐restriction period preceded the experimental trial, during which nutritional ad libitum access was reinstated through the concurrent presentation of 1% sucrose and distilled water. Volumetric consumption parameters were gravimetrically quantified through pre‐ and post‐test mass differential measurements over a 12 h testing interval. Sucrose preference indices were calculated using the following standardized formula:

$$\begin{array}{c} \text{Sucrose preference}\;\left(\%\right)=\\ \;\left[\text{sucrose intake}/\left(\text{sucrose intake}+\text{water intake}\right)\right]\times 100\% \end{array}$$

with measurements conducted under controlled environmental conditions to minimize confounding variables.

### 2.4 | Stereotaxic Surgery

Mice were anesthetized using isoflurane vaporization and immobilized in a stereotaxic apparatus (RWD Life Science Co. Ltd., Shenzhen, China) with corneal protection maintained through the application of ophthalmic erythromycin ointment. Craniometric measurements were referenced as the bregma landmarks. To investigate the mechanism of GPR35, a GPR35‐specific knockdown lentivirus (GPR35^KD^), GPR35 overexpression lentivirus (GPR35^OE^), or control virus (GPR35^Scr1^ or GPR35^Scr2^) was delivered into the ACC of mice using a 10‐μL precision microsyringe (Gaoge, Shanghai, China) coupled to a microelectrode, with infusion parameters maintained at 50 nL/min via a microprocessor‐controlled injection system (Kd Scientific, Holliston, MA, USA). Post‐infusion stabilization was achieved through a 10 min dwell period to facilitate viral dispersion. Bregma coordinates for ACC targeting were standardized at anterior posterior +1.00 mm, medial lateral ±0.25 mm, and dorsal ventral −1.90 mm. After needle retraction, the craniotomy sites were sealed with sterile bone wax. The CCI mice was established after a 7 day recovery period to permit optimal viral expression, then ACC tissues were collected for sequencing analysis.

### 2.5 Western Blotting

Mice brain protein extracts were separated using 10% SDS‐PAGE and subsequently transferred to a BioTrace NT nitrocellulose membrane (Washington, USA, Pall, #66485). Following transfer, the membrane was blocked for 2 h at room temperature (23°C ± 2°C) using 5% non‐fat milk or bovine serum albumin (BSA) and then incubated with primary antibodies overnight at 4°C. Rabbit anti‐GPR35 (Novus Biologicals, USA; Cat#NBP2‐24640; 1:1000), mouse anti‐Nr4a1 (Santa Cruz Biotechnology, USA; Cat#sc‐365113; 1:1000), rabbit anti‐p‐PI3K (Affinity Biosciences, China; Cat#AF3241; 1:1000), mouse anti‐PI3K (Santa Cruz Biotechnology; Cat#sc‐1637; 1:1000), mouse anti‐p‐AKT (Santa Cruz Biotechnology; Cat#sc‐81433; 1:1000), mouse anti‐AKT (Santa Cruz Biotechnology; Cat#sc‐81434; 1:1000), and rabbit anti‐β‐actin (Beyotime Biotechnology, China; Cat#AF5006; 1:500). After three washes with tris buffered saline tween, the membranes were incubated with the appropriate secondary antibodies for 12 h. The protein bands were visualized using a chemiluminescent HRP substrate (Merck‐Millipore, #WBKLS0050) and imaged using a ChemiDoc XRS system (Bio‐Rad). Densitometric analysis was performed using ImageJ software version 1.8.0, with protein expression levels normalized to β‐actin as an internal loading control.

### 2.6 RT‐qPCR

Total RNA was extracted from the tissue using TRIzol reagent and quantified using spectrophotometry. RNA integrity was verified by ensuring all samples had A260/A280 ratios between 1.8 and 2.0 (NanoDrop, Thermo Fisher Scientific). For each sample, 1 μg of total RNA was reverse‐transcribed in a 20‐μL reaction using Hifair III SuperMix (Yeasen, China) with the manufacturer's recommended thermal profile (25°C for 5 min, 55°C for 15 min, 85°C for 5 min). Quantitative PCR was performed in triplicate on a Bio‐Rad CFX Connect system using SYBR Green chemistry (Accurate Biology, China). The two‐step thermal cycling protocol included a holding stage (95°C for 30 s) and 40 cycles of amplification (95°C for 5 s, 60°C for 30 s), followed by a melt curve stage to confirm amplicon specificity. Gene expression was normalized to GAPDH and calculated via the 2^−ΔΔCt^ method. The oligonucleotide primer sequences are listed in Table S1. All reactions were performed in triplicate to ensure experimental reproducibility.

### 2.7 Immunofluorescence Staining

Mice were anesthetized and subjected to transcardial perfusion with saline followed by 4% paraformaldehyde. Brain tissues were collected, fixed overnight in 4% paraformaldehyde, and cryoprotected in 30% sucrose at 4°C until the tissues settled to the bottom of the container. Serial sections (6‐μm thick) were cut using a cryostat and immunostained with the following primary antibodies: mouse anti‐NeuN (Cell Signaling Technology, USA; Cat#94403S; 1:200), rabbit anti‐GPR35 (Novus Biologicals; Cat#NBP2‐24640; 1:200), rabbit anti‐CD68 (Abcam, USA; Cat#ab125212; 1:200), and mouse anti‐IBA1 (Thermo Fisher Scientific, USA; Cat#GT10312; 1:100). Sections were incubated at room temperature for 1 h with a solution containing 0.1% Triton X‐100 and 3% BSA. The sections were then incubated with primary antibodies overnight at 4°C. After three washes with phosphate buffer solution (PBS), the sections were incubated for 2 h at room temperature with the following secondary antibodies: DyLight 594‐conjugated goat anti‐rabbit IgG (EarthOx, USA; Cat#E032420‐01; 1:500) and DyLight 488‐conjugated goat anti‐mouse IgG (EarthOx; Cat#E032210‐01; 1:500). Nuclear counterstaining was performed using DAPI (Beyotime Biotechnology; Cat#P0131‐25 mL), followed by PBS rinsing. All images were captured and analyzed using a confocal microscope (LSM800, ZEISS, Germany).

### 2.8 Statistical Analysis

All data are presented as mean ± SEM. Pearson correlation analysis was performed to assess the relationship between GPR35 expression and McGill pain score. Statistical analyses were performed using GraphPad Prism version 9. The Shapiro–Wilk test was used to assess whether the data followed a Gaussian distribution, followed by *t*‐tests, one‐way ANOVA, or two‐way ANOVA as appropriate. Data that did not exhibit a normal/Gaussian distribution were analyzed using non‐parametric equivalents with post hoc tests for pairwise comparisons. A *p* < 0.05 was considered statistically significant.

## 3 Results

### 3.1 GPR35 Expression Was Increased in Chronic Pain Patients and CCI Mice

**FIGURE 1:**
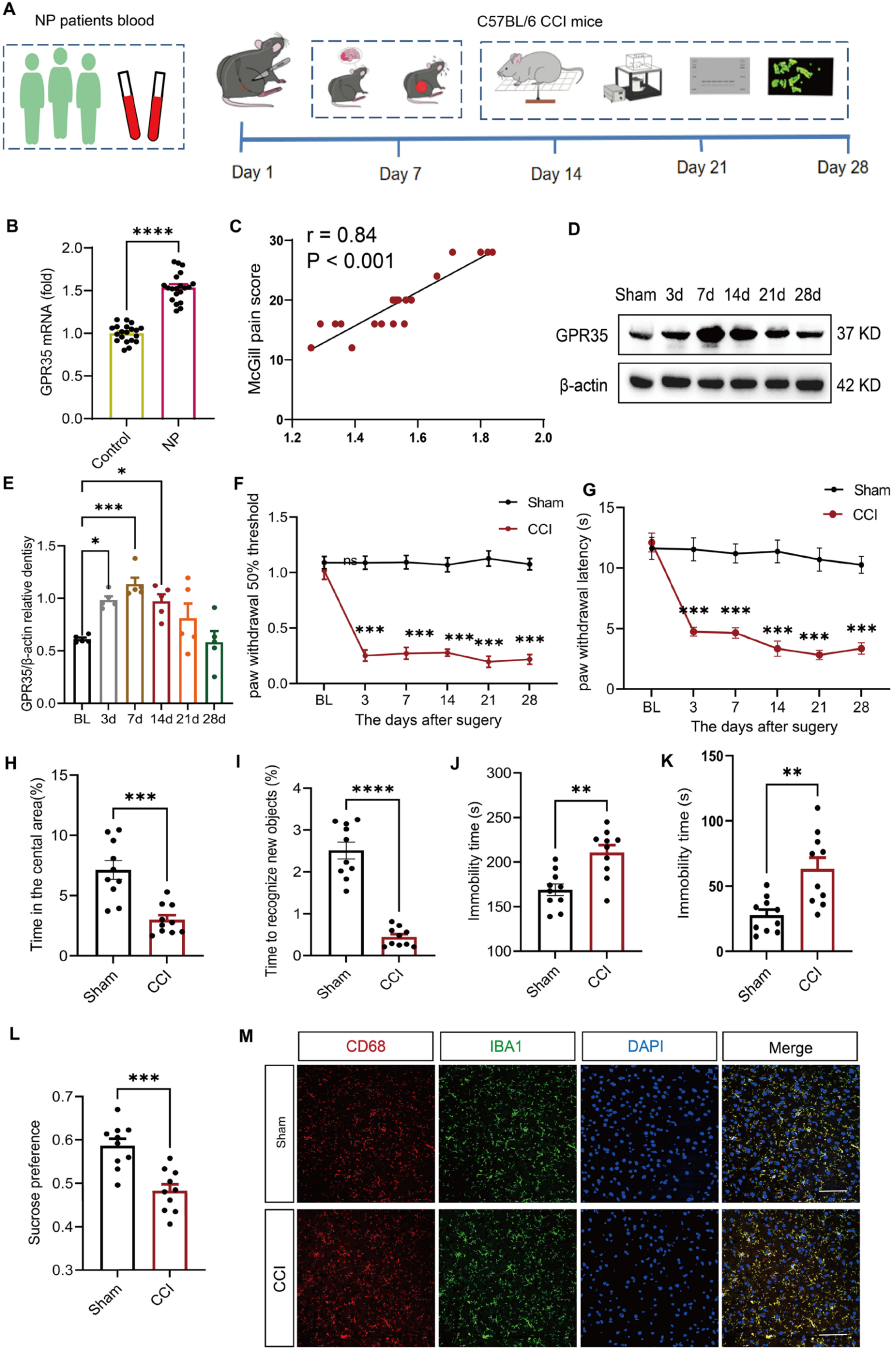
GPR35 expression was increased in patients with chronic pain and CCI mice. (A) Simplified experimental flowchart. (B) Blood GPR35 levels were increased in patients with neuropathic pain (*n =* 20) compared with that in healthy controls. *n =* 20. ****p < 0.001. (C) Pearson correlation coefficient was used to analyze the correlation between GPR35 expression and McGill pain score. (D) Relative gene expression of GPR35 in the ACC of mice 3, 7, 14, 21, and 28 days after CCI establishment and sham operation group. *n =* 5. (E) Density expression of GPR35/β‐actin. *n =* 5, *p < 0.05, ***p < 0.001. (F) PWT of mice at 1 day before surgery and 3, 7, 14, 21, and 28 days after surgery. *n =* 10, ***p < 0.001. (G) PWL of mice at 1 day before surgery and 3, 7, 14, 21, and 28 days after surgery. *n =* 10, ***p < 0.001. (H) Percentage of time spent in the central area of mice during the open field test, OFT. *n =* 10, ***p < 0.001. (I) The percentage of time spent by mice to recognize novel objects in novel object recognition. *n =* 10, ****p < 0.001. (J) Tail suspension test, TST. *n =* 10, **p < 0.01. (K) Forced swimming test, FST. *n =* 10, **p < 0.01. (L) Sucrose preference. *n =* 10, ***p < 0.001. (M) Fluorescence expression of co‐staining of CD68^+^ and IBA1^+^ in the ACC region of mice, scale bar: 20 μm. *n* = 4. CCI, chronic constriction injury; NP, neuropathic pain; PWL, thermal paw withdrawal latency; PWT, mechanical paw withdrawal threshold.

The flowchart of the experiment is shown in Figure 1A. To elucidate the functional involvement of GPR35 in NP pathogenesis, we initially quantified GPR35 expression profiles in whole blood samples obtained from healthy control human participants (*n =* 20) and human patients with NP diagnosed with sciatic nerve compression secondary to lumbar disc herniation (*n =* 20), complemented by McGill Pain Questionnaire evaluation. Quantitative analysis demonstrated a significant elevation in whole blood GPR35 concentrations in patients with NP relative to healthy controls (*p <* 0.001) (Figure 1B). Subsequent linear regression modeling identified a robust positive correlation between circulating GPR35 levels and subjective pain intensity scores (*r* = 0.84, *p <* 0.001; Figure 1C). The demographic and clinical characteristics of the participants are shown in Table S2.

In parallel murine studies, the temporal expression patterns of GPR35 in the ACC were examined following CCI. Western blotting revealed a characteristic temporal profile: initial upregulation at 3 days post‐surgery and maximal expression at day 7 (*p <* 0.001), followed by a gradual return to baseline levels (Figure 1D,E). Behavioral phenotyping confirmed the successful CCI mice induction, as indicated by sustained mechanical allodynia (Figure 1F) and thermal hyperalgesia (Figure 1G). Furthermore, CCI mice exhibited characteristic neuropsychiatric comorbidities, including anxiety‐like behaviors (quantified by reduced central zone exploration time in the OFT) and depression‐like phenotypes (manifested as increased immobility duration in tail suspension tests and diminished sucrose preference) (Figure 1H–L). Immunofluorescence analysis demonstrated a significant increase in CD68^+^/IBA1^+^ double‐positive cells in the model group compared with that in the sham group (*p <* 0.05; Figure 1M), indicating enhanced microglial activation. Furthermore, GPR35 in the ACC was predominantly localized to neurons and microglia, as evidenced by colocalization studies (Figure S1).

### 3.2 GPR35 Knockdown Amplifies Nociceptive Hypersensitivity, Affective Dysregulation, and Neuroinflammatory Responses in CCI Mice

**FIGURE 2:**
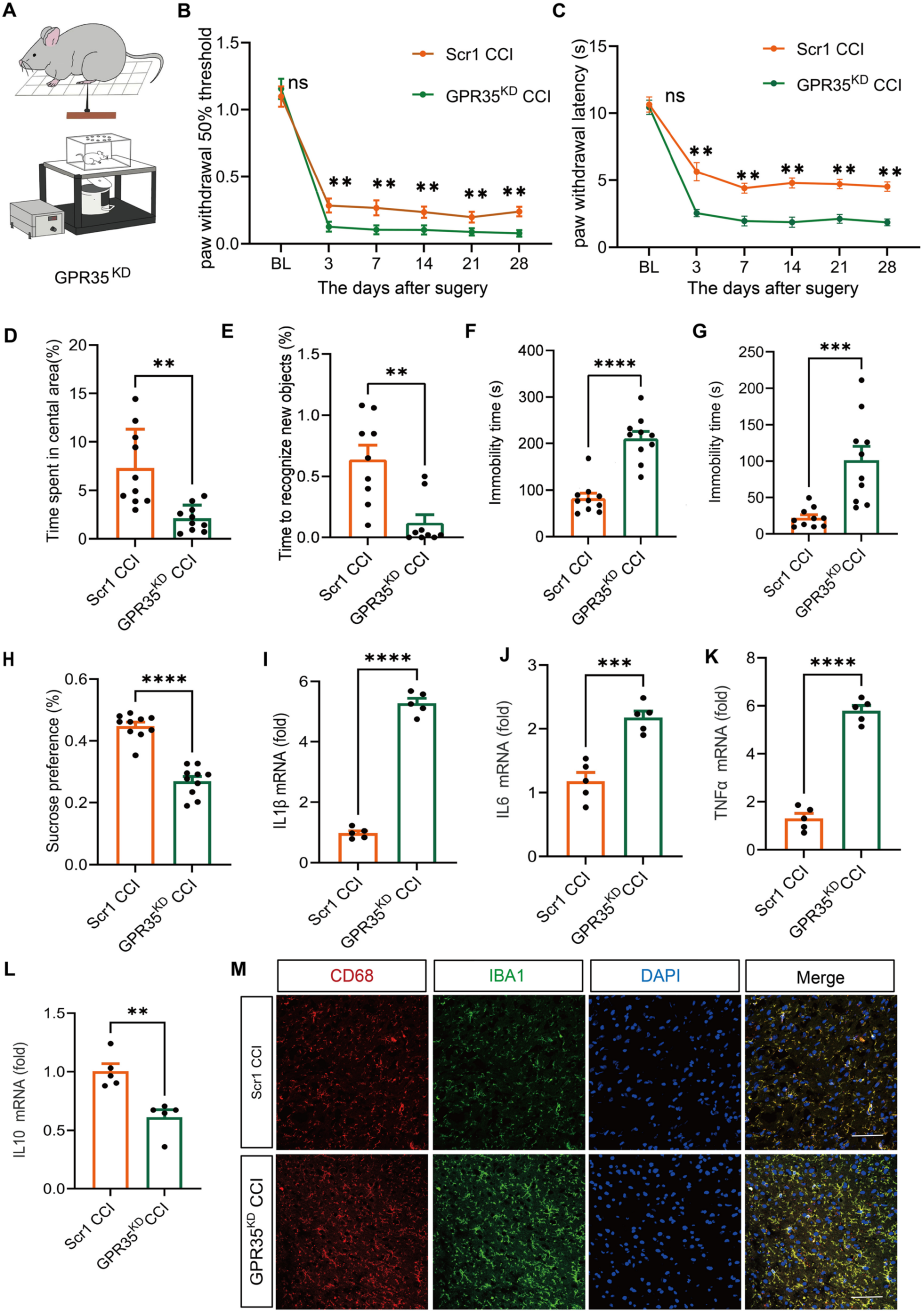
Downregulation of GPR35 aggravated pain and depression‐like behaviors in CCI mice. (A) Schematic diagram of intra‐ACC GPR35^KD^ virus injection into C57BL/6 mice. (B) PWT of mice at 1 day before surgery and 3, 7, 14, 21, and 28 days after surgery. *n =* 10, **p < 0.01. (C) PWL of mice at 1 day before surgery and 3, 7, 14, 21, and 28 days after surgery. *n =* 10, **p < 0.01. (D) Percentage of time spent in the central area during the open field test. *n =* 10, **p < 0.01. (E) The percentage of time spent by mice to recognize novel objects in novel object recognition. *n =* 10, **p < 0.01. (F) The tail suspension test, TST. *n =* 10, ****p < 0.001. (G) Forced swimming test, FST. *n =* 10, ***p < 0.001. (H) Preference for sugar water. *n =* 10, ****p < 0.001. (I–M) mRNA in ACC expression (IL1β, IL6, TNFα, and IL10). *n =* 5, **p < 0.01, ***p < 0.001, ****p < 0.001. (M) Fluorescence expression of co‐staining of CD68^+^ and IBA1^+^ in the ACC region of mice, scale bar: 20 μm. *n =* 4. ACC, anterior cingulate cortex; CCI, chronic constriction injury; PWT, mechanical paw withdrawal threshold; PWL, thermal paw withdrawal latency.

To investigate the mechanism of GPR35 in NP, we delivered a GPR35‐specific knockdown lentivirus (GPR35^KD^) or control virus (GPR35^Scr1^) into the ACC of mice via stereotaxic injection (Figures 2A and S2A). Quantitative western blotting analysis at 7 days post‐injection validated the marked attenuation of GPR35 protein levels in the bilateral ACC of GPR35^KD^ mice relative to scrambled vector‐treated controls (*p* < 0.001; Figure S2B,C).

Behavioral assessments revealed that GPR35^KD^ mice exhibited more pronounced pain hypersensitivity, reduced mechanical paw withdrawal threshold (PWT; *p <* 0.01; Figure 2B), and shortened thermal paw withdrawal latency (PWL; *p <* 0.01; Figure 2C) compared with those of controls. Additionally, GPR35^KD^ exacerbated CCI‐induced emotional disturbances, as evidenced by reduced central zone dwell time in the OFT (*p <* 0.01; Figure 2D), impaired NOR (*p <* 0.01; Figure 2E), prolonged immobility time in the TST (*p <* 0.001; Figure 2F), increased immobility time in the FST (*p <* 0.001; Figure 2G), and decreased SPT (*p <* 0.001; Figure 2H).

Mechanistic studies demonstrated that GPR35^KD^ significantly altered the balance of inflammatory factors in the ACC; pro‐inflammatory mediators, including IL1β, IL6, and TNFα (*p <* 0.001; Figure 2I–K) were upregulated and the anti‐inflammatory cytokine, IL10, was downregulated (*p <* 0.01; Figure 2L). Immunofluorescence analysis demonstrated enhanced microglial activation in GPR35^KD^, as evidenced by an increase in the co‐staining of CD68^+^ and IBA1^+^ (*p* < 0.05; Figure 2M) relative to those of the CCI controls. Collectively, these indicate that GPR35 deficiency in the ACC potentiates nociceptive hypersensitivity and affective dysregulation through the amplification of neuroinflammatory signaling pathways, highlighting its critical role in modulating pain‐associated neuroimmune responses.

### 3.3 Overexpression of GPR35 Alleviates Pain Depression‐Like Behavior and Neuroinflammation in CCI Mice

**FIGURE 3:**
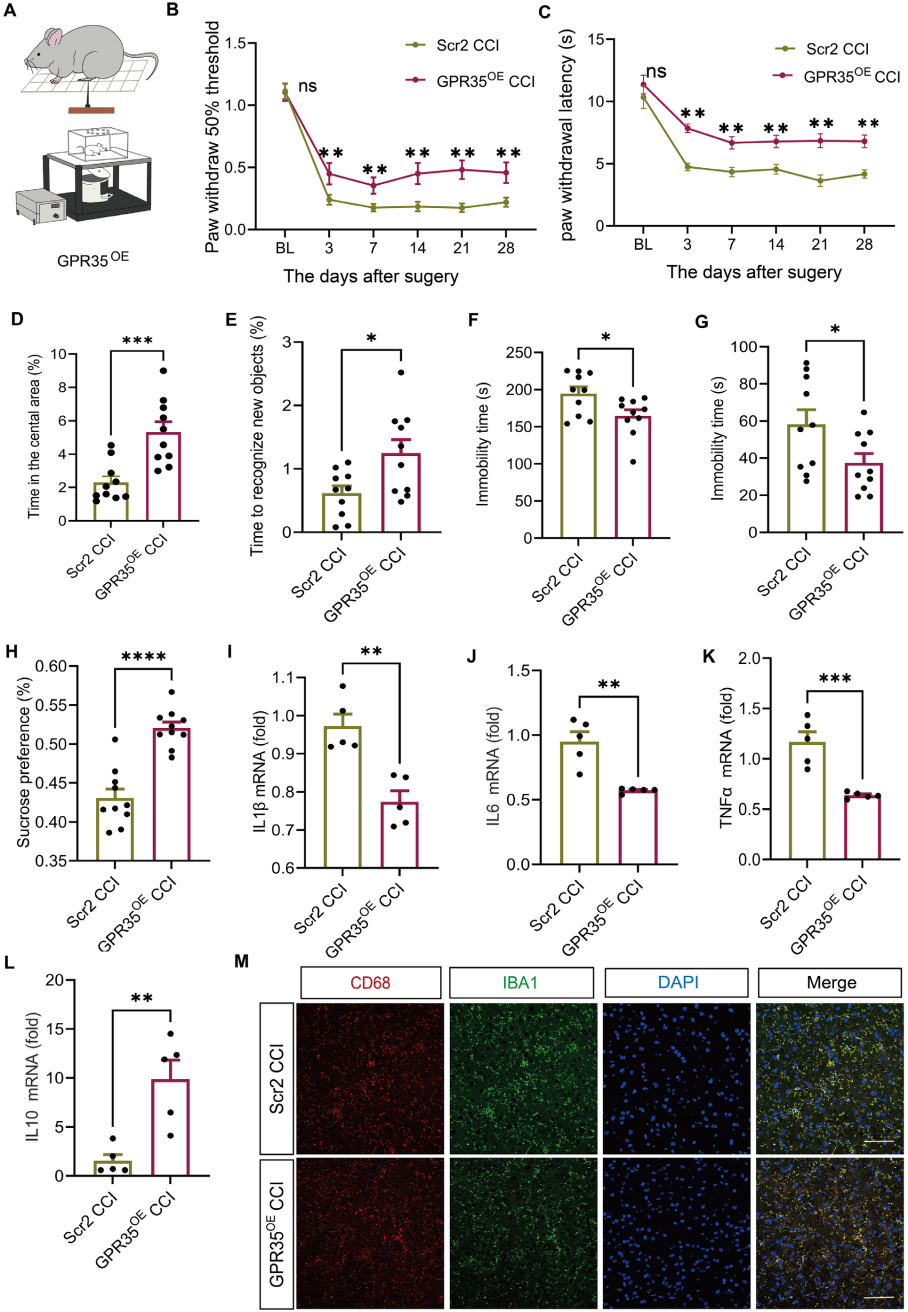
Overexpression of GPR35 reduced pain and depression‐like behaviors in CCI mice. (A) Schematic diagram of intra‐ACC GPR35^OE^ virus injection into C57BL/6 mice. (B) PWT of mice at 1 day before surgery and 3, 7, 14, 21, and 28 days after surgery. *n =* 10, **p < 0.01. (C) PWL of mice at 1 day before surgery and 3, 7, 14, 21, and 28 days after surgery. *n =* 10, **p < 0.01. (D) Percentage of time spent in the central area during the open field test. *n =* 10, ***p < 0.001. (E) The percentage of time spent by mice to recognize novel objects in novel object recognition. *n =* 10, *p < 0.05. (F) The tail suspension test, TST. *n =* 10, *p < 0.05. (G) Forced swimming test, FST. *n =* 10, *p < 0.05. (H) Sucrose preference. *n =* 10, ****p < 0.001. (I–L) mRNA expression in ACC expression (IL1β, IL6, TNFα, and IL10). *n =* 5, **p < 0.01, ***p < 0.001. (M) Fluorescence expression of co‐staining of CD68^+^ and IBA1^+^ in the ACC region of mice. Scale bar: 20 μm. *n =* 4. ACC, anterior cingulate cortex; CCI, chronic constriction injury; PWL, thermal paw withdrawal latency; PWT, mechanical paw withdrawal threshold.

To further validate GPR35 function, we delivered a GPR35 overexpression lentivirus (GPR35^OE^) or control virus (GPR35^Scr2^) into the ACC of mice via stereotaxic injection (Figures 3A and S3A). One week post‐injection, western blotting confirmed a significant upregulation of GPR35 protein expression in the ACC of GPR35^OE^ mice compared with that in controls (*p <* 0.01; Figure S3B,C).

Behavioral assessments demonstrated that GPR35^OE^ significantly ameliorated pain hypersensitivity, increased PWT (*p <* 0.01; Figure 3B) and prolonged PWL (*p <* 0.01; Figure 3C). Concurrently, GPR35^OE^ alleviated CCI‐induced emotional disturbances, as manifested by an increased central zone dwell time in the OFT (*p <* 0.001; Figure 3D), improved NOR (*p <* 0.05; Figure 3E), reduced immobility time in the TST (*p <* 0.05; Figure 3F), decreased immobility time in the FST (*p <* 0.05; Figure 3G), and elevated the sucrose preference rate (*p <* 0.001; Figure 3H).

Mechanistic investigations revealed that GPR35^OE^ normalized the inflammatory cytokine balance in the ACC by downregulating pro‐inflammatory factors IL1β, IL6, and TNFα (*p <* 0.01; Figure 3I–K) and upregulating the anti‐inflammatory factor, IL10 (*p <* 0.01; Figure 3L). Immunofluorescence quantification revealed reduced microglial activation, with decreased co‐staining for CD68^+^ and IBA1^+^ (*p <* 0.05; Figure 3M). Collectively, these results demonstrate that GPR35^OE^ mitigates nociceptive hypersensitivity and affective dysregulation in CCI mice by suppressing neuroinflammatory signaling pathways, underscoring its therapeutic potential in modulating pain‐related neuroimmune mechanisms.

### 3.4 Nr4a1 Mediates GPR35 Regulation of Neuropathic in NP

**FIGURE 4:**
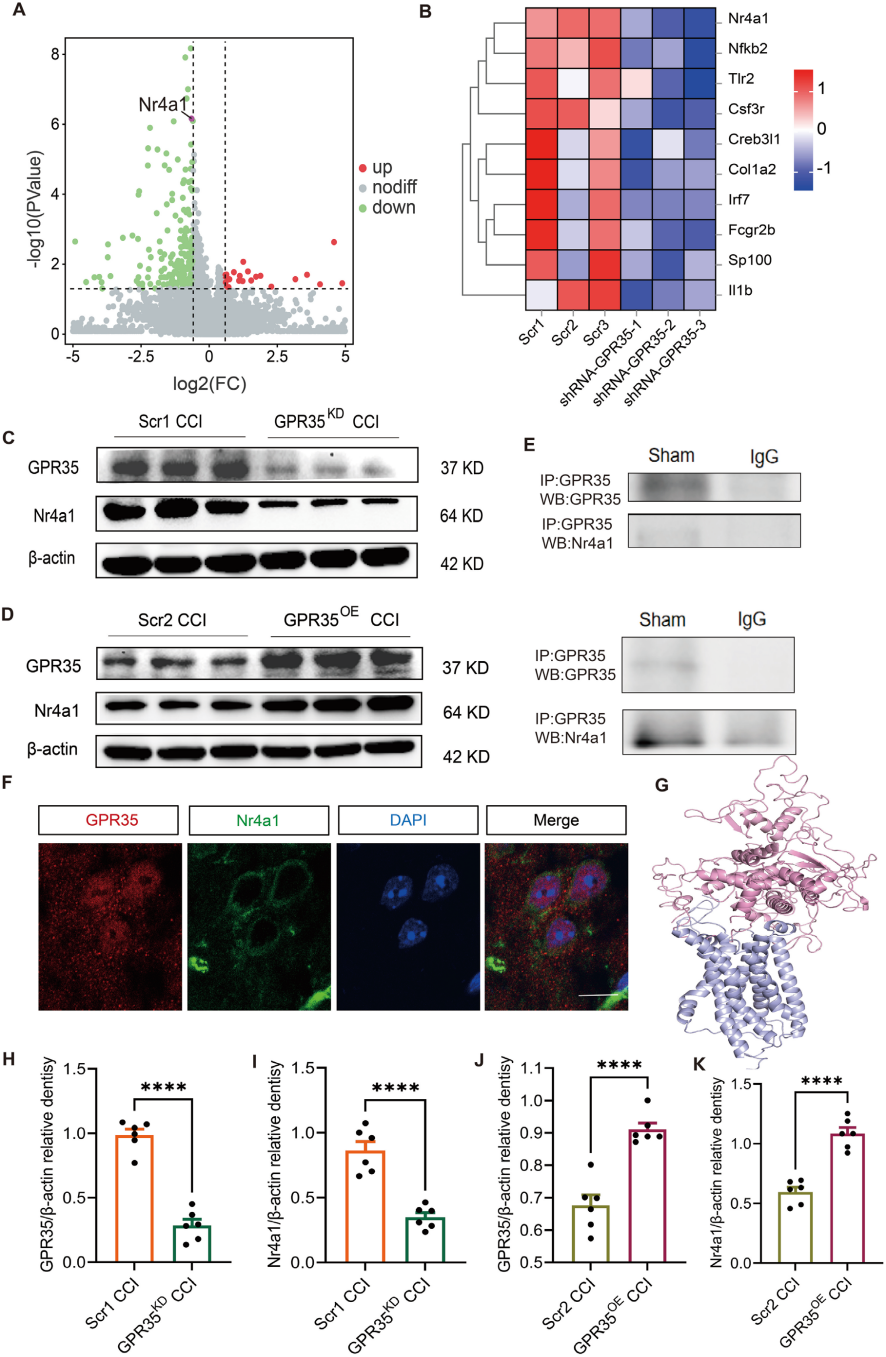
Screening and validation of Nr4a1 as a downstream molecule of GPR35. (A) Volcano plot showing relative expression of interacting pathway genes after GPR35 knockdown. *n =* 3 mice/group. (B) Heatmap of relative expression of interacting pathway genes after GPR35 knockdown. *n =* 3. (C, H, I) Significant downregulation of Nr4a1 expression in CCI mice after GPR35 knockdown. *n =* 6, ****p < 0.001. (D, J, K) Significant upregulation of Nr4a1 expression in CCI mice after GPR35 overexpression. *n =* 6, ****p < 0.001. (E) Co‐immunoprecipitation of GPR35 and Nr4a1. (F) Immunofluorescence colocalization of GPR35 (red) and Nr4a1 (green). Scale bar: 40 μm. *n =* 4. (G) Molecular docking model of GPR35 and Nr4a1 interaction. CCI, chronic constriction injury.

To elucidate the mechanism of GPR35, we performed RNA‐seq to analyze the transcriptomic differences in the ACC between GPR35^KD^ and control (GPR35^Scr1^) mice. RNA‐seq revealed that Nr4a1 expression was significantly reduced after GPR35^KD^, the volcano plot and heatmap are shown in Figure 4A,B. Western blotting results demonstrated that GPR35^KD^ significantly downregulated Nr4a1 protein expression (*p <* 0.05), whereas GPR35^OE^ upregulated Nr4a1 levels (*p <* 0.001; Figure 4C,D,H–K). Co‐immunoprecipitation assays and immunofluorescence colocalization confirmed a direct interaction between GPR35 and Nr4a1 (Figure 4E,F). Molecular docking simulations predicted an interaction between GPR35 and Nr4a1, as shown in Figure 4G.

**FIGURE 5:**
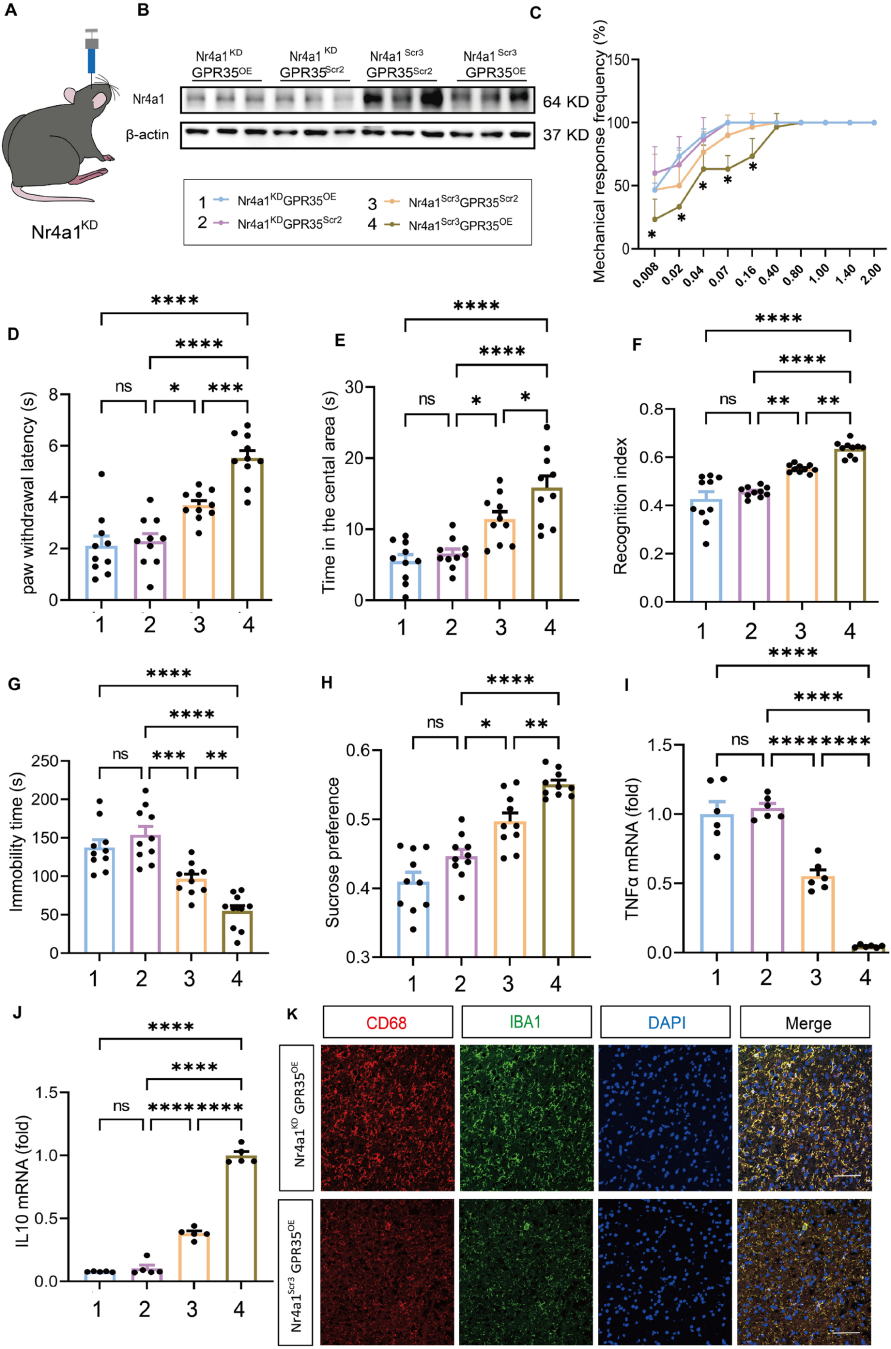
Knockdown of Nr4a1 abolished the pain‐relieving effect of GPR35. (A) Schematic diagram of intra‐ACC Nr4a1^KD^ virus injection into C57BL/6 mice. (B) Validation of the effect of Nr4a1 knockdown in mice showed that knockdown was significant. *n =* 6. (C) Knockdown of Nr4a1 abolished the protective effect of GPR35 overexpression on mechanical pain in mice. *n =* 10. *p < 0.05. (D) Nr4a1 knockdown abolished the protective effect of GPR35 overexpression on thermal pain in mice. *n =* 10. (E–H) Nr4a1 knockdown abolished the protective effect of GPR35 overexpression on depression‐like behaviors in mice. *n =* 10. (I–K) Nr4a1 knockdown abolished the protective effect of GPR35 overexpression on inflammation (TNFα, IL10, and CD68). *n =* 6. Scale bar: 20 μm. *p < 0.05, **p < 0.01, ***p < 0.001, ****p < 0.001, respectively.

To validate the pivotal role of Nr4a1 in the GPR35 regulatory network, we knocked down Nr4a1 in GPR35^OE^ or control (GPR35^Scr2^) mice (Figure 5A). Western blotting confirmed efficient Nr4a1 knockdown (Figure 5B). As mentioned earlier, behavioral and molecular biological evaluations after overexpression of GPR35 showed that the pain, depression, and neuroinflammation in mice were significantly alleviated. However, Nr4a1 knockdown reversed the protective effects of GPR35^OE^ was reversed (*p <* 0.05; Figure 5C–H).

Mechanistic studies indicated that GPR35^OE^ significantly reduced pro‐inflammatory cytokines TNFα (*p <* 0.001; Figure 5I) and IL6 (*p <* 0.05; Figure S4A) and upregulated anti‐inflammatory IL10 (*p <* 0.001; Figure 5J). When Nr4a1 was knocked down, the activation level of microglia increased, which was confirmed by increased co‐staining of CD68^+^ and IBA1^+^ (Figure S4B). After GPR35 overexpression, the activation level of microglia decreased, as evidenced by the reduction in the co‐staining of CD68^+^ and IBA1^+^, which was reversed by Nr4a1 knockdown (*p <* 0.05; Figure 5K). These findings confirm that Nr4a1 is a critical downstream effector mediating neuroprotective effects of GPR35.

### 3.5 GPR35 Regulates Pain and Depression‐Like Behaviors via the PI3K/AKT Signaling Pathway

**FIGURE 6:**
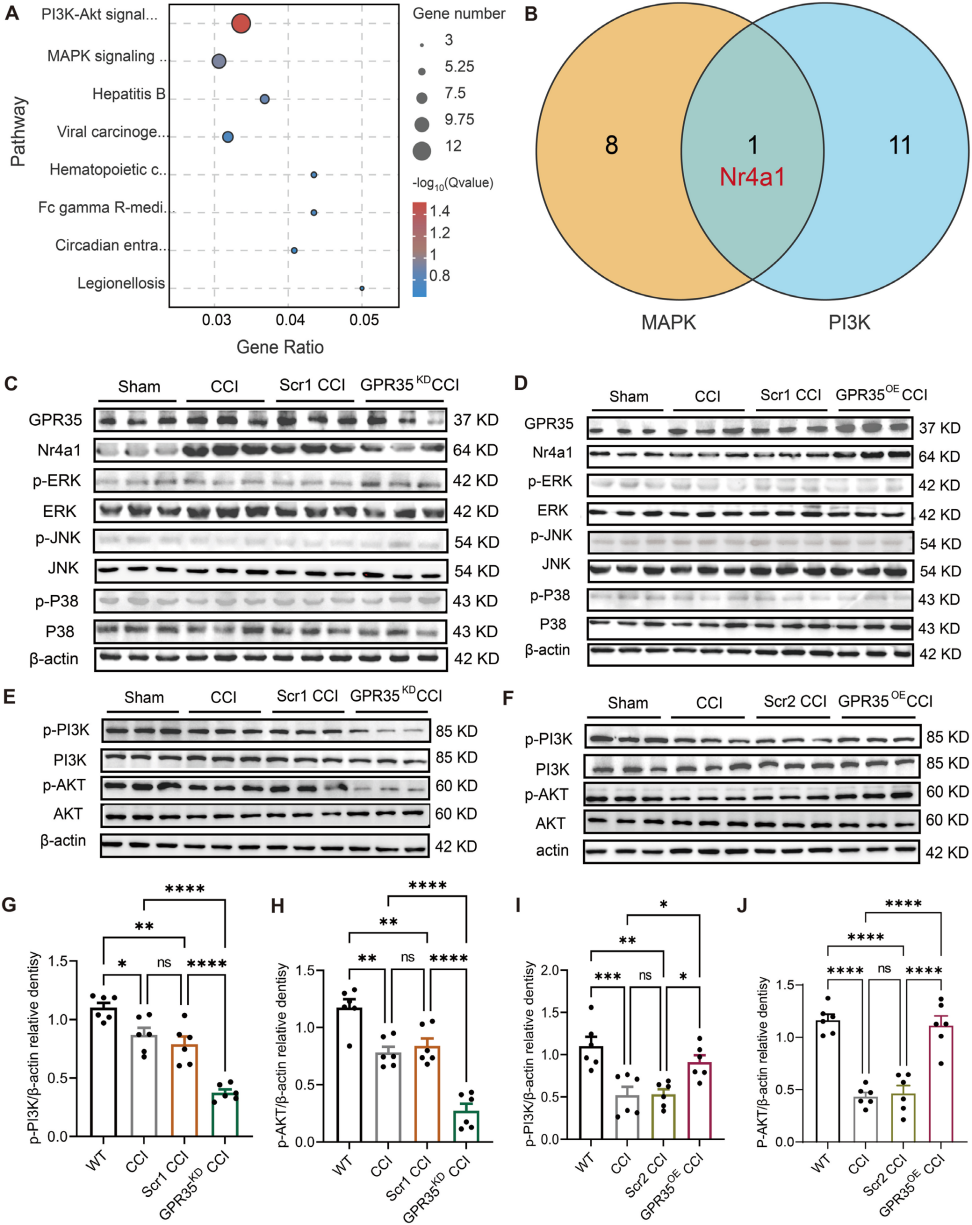
GPR35 regulates pain‐and depression‐like behaviors in mice through the Nr4a1/PI3K/AKT pathway. (A) Transcriptome sequencing of the GPR35^KD^ model group and GPR35^Scr1^ CCI mice revealed eight of the most significantly enriched CCI inflammation‐related pathways. *n =* 3. (B) Venn diagram of the intersection of the two most significantly enriched pathways (MAPK and PI3K/AKT pathways), and the common gene where the two pathways intersect is Nr4a1. *n =* 3. (C) There were no significant differences in the expression of ERK, JNK, p38 or their phosphorylated proteins in the MAPK pathway after GPR35 knockdown in CCI mice. *n =* 6. (D) No significant differences were observed in the expression of ERK, JNK, p38 and their phosphorylated proteins in the MAPK pathway after GPR35 overexpression in CCI mice. *n =* 6. (E, G, H) Changes in the expression of PI3K, AKT and their phosphorylated proteins after GPR35 knockdown in CCI mice. *n =* 6. *p < 0.05, **p < 0.01, ****p < 0.001, respectively. (F, I, J) Changes in the expression of PI3K, AKT, and phosphorylated proteins after GPR35 overexpression in the CCI mice. *n =* 6. *p < 0.05, **p < 0.01, ***p < 0.001, ****p < 0.001, respectively. ACC, anterior cingulate cortex; CCI, chronic constriction injury.

Differential expression analysis revealed that GPR35^KD^ significantly altered eight inflammation‐related signaling pathways (*p <* 0.01), with the MAPK and PI3K/AKT pathways being the most prominent (Figure 6A). Nr4a1 was identified as a common regulatory target in both pathways (Figure 6B).

To corroborate the RNA‐seq findings, we conducted quantitative assessments of both the expression and phosphorylation of core MAPK signaling components. Neither GPR35 knockdown nor overexpression significantly affected the MAPK pathway (Figure 6C,D). However, the PI3K/AKT pathway exhibited significant changes; p‐PI3K and p‐AKT expression was downregulated in GPR35^KD^ mice (*p <* 0.05) and upregulated in GPR35^OE^ mice (*p <* 0.05) compared to that in controls (Figure 6E,F). Quantitative western blotting analysis of p‐PI3K and p‐AKT is shown in Figure 6G–J, and data for other non‐significantly altered proteins are shown in Figure S5. Collectively, these findings demonstrate that GPR35 predominantly modulates nociceptive hypersensitivity and affective dysregulation in CCI mice through selective regulation of the PI3K/AKT signaling axis.

### 3.6 GPR35 Agonist L‐Kyna Alleviates Pain Hypersensitivity and Depression‐Like Behaviors in CCI Mice

**FIGURE 7:**
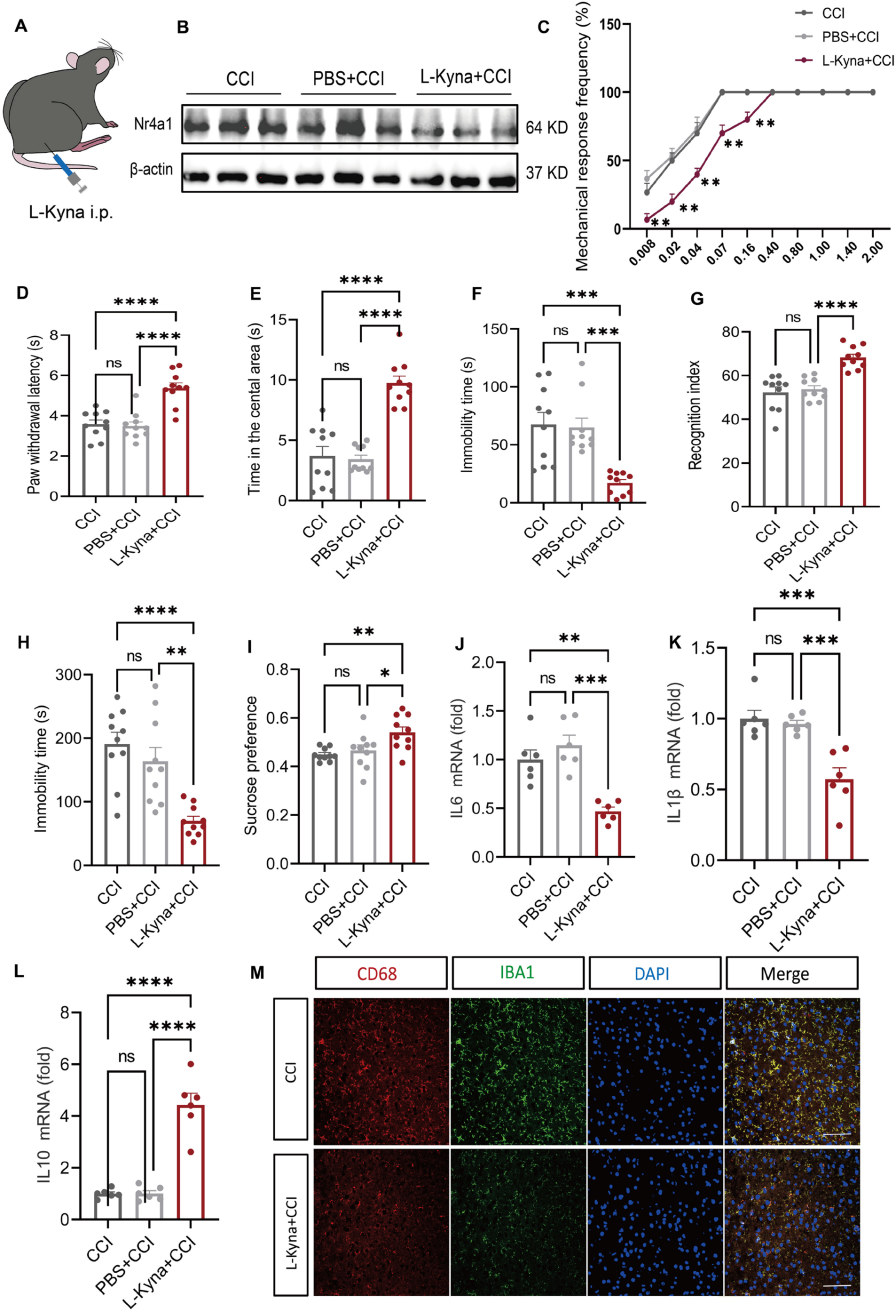
L‐Kyna, an agonist of GPR35, reduced neuroinflammation in mice. (A) Schematic diagram of intraperitoneal injection of L‐Kyna into C57BL/6 mice. (B) Nr4a1 expression was significantly decreased after L‐Kyna injection. *n =* 5. (C) L‐Kyna injection significantly increased the mechanical pain threshold in mice. *n =* 9, **p < 0.01. (D) L‐Kyna significantly increased the heat pain threshold in mice after injection. *n =* 9, ****p < 0.001. (E) Percentage of time spent in the central area during the open‐field test. *n =* 9, ****p < 0.001. (F) Tail suspension test, TST. *n =* 10, ***p < 0.01. (G) Percentage of time spent recognizing novel objects. *n =* 9, ****p < 0.001. (H) Forced swimming test, FST. *n =* 10, **p < 0.01, ****p < 0.001. (I) Sucrose preference. *n =* 10, *p < 0.05, **p < 0.01. (J–M) Injection of L‐Kyna regulated the levels of inflammatory factors (IL6, IL1β, and IL10) and the number of microglia co‐stained with CD68^+^ and IBA1^+^. *n* = 6. Scale bar: 20 μm. **p < 0.01, ***p < 0.001, ****p < 0.001, respectively. L‐Kyna, L‐kynurenine.

To investigate the therapeutic potential of L‐Kyna, we administered intraperitoneal injections of L‐Kyna (Bioresearch products; Cat#K8625; 10 mg/kg) for seven consecutive days after CCI mice establishment, with control mice receiving an equal volume of PBS (Figure 7A).

**FIGURE 8:**
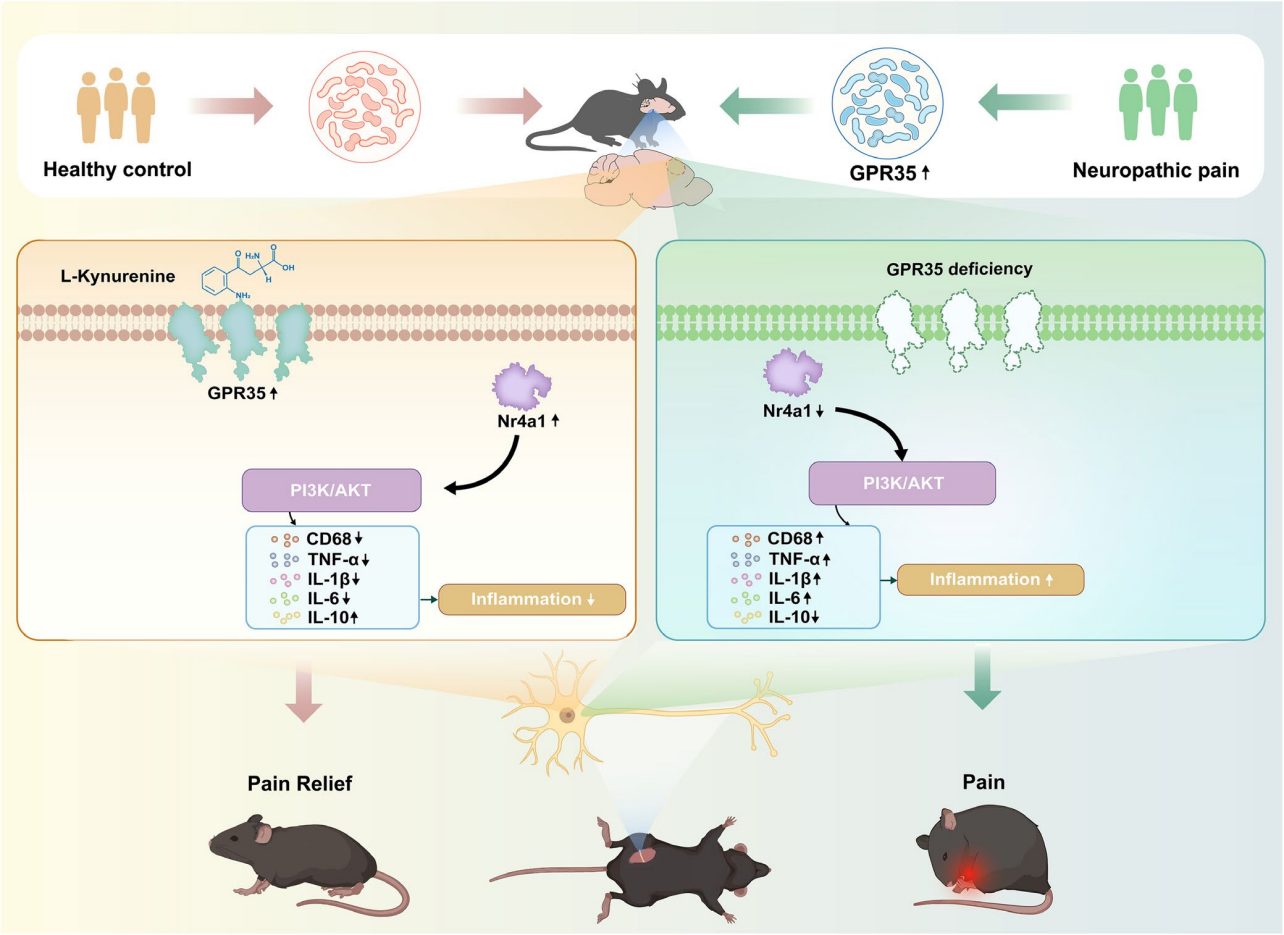
The model describes how GPR35 in the ACC region controls inflammation through the Nr4a1/PI3K/AKT signaling pathway, thereby mitigating the critical role of NP. Sciatic nerve injury led to a significant increase in the expression of GPR35 in the ACC region of mice. This increased interaction with Nr4a1 can activate the PI3K/AKT signaling pathway, thereby reducing neuroinflammation and playing a role in alleviating hyperalgesia and depressive behavior in mice. ACC, anterior cingulate cortex.

Mechanistic studies showed that L‐Kyna significantly downregulated Nr4a1 expression in the ACC of CCI mice (*p <* 0.05; Figure 7B). Behavioral assessments revealed that L‐Kyna significantly improved PWT (*p <* 0.01; Figure 7C) and prolonged PWL (*p <* 0.001; Figure 7D). Depression‐like behaviors were alleviated, manifested as an increase in central zone dwell time (*p <* 0.001; Figure 7E), reduced immobility time in TST (*p <* 0.01; Figure 7F), enhanced NOR (*p <* 0.001; Figure 7G), decreased immobility in FST (*p <* 0.01; Figure 7H), and elevated SPT (*p <* 0.05; Figure 7I). Inflammatory cytokine assays demonstrated that L‐Kyna significantly reduced IL6 and IL1β (*p <* 0.05) levels and upregulated IL10 (*p <* 0.01; Figure 7J–L). Immunofluorescence staining further confirmed that L‐Kyna decreased the number of co‐stained CD68^+^ and IBA1^+^ microglia in the ACC (*p <* 0.05) (Figures 7M and S4C). These findings indicate that L‐Kyna exerts analgesic and antidepressant effects by activating the GPR35‐Nr4a1 signaling axis. Figure 8 shows the schematic illustration of the GPR35‐mediated neurogenic inflammatory cascade in the ACC and consequent progression of NP.

## 4 Discussion

This study elucidates the critical regulatory role of GPR35 in NP development. Compared with the healthy control participants, the expression of GPR35 in patients with NP was significantly increased. In animal experiments, sciatic nerve injury led to a significant increase in the expression of GPR35 in the ACC. The increased interaction with Nr4a1, a key downstream effector of GPR35, can activate the PI3K/AKT signaling pathway, thereby reducing neuroinflammation and playing a role in alleviating hyperalgesia and depressive behavior in mice.

Clinical sample analysis demonstrated significant upregulation of GPR35 expression in whole blood from patients with NP. Linear regression analysis demonstrated a robust positive correlation between GPR35 expression and subjective pain intensity scores, as assessed using the McGill Pain Questionnaire (Figure 1A,B). These findings suggest that GPR35 is involved in NP pathogenesis and has potential as a biomarker of NP. In the CCI mice, we observed dynamic changes in GPR35 expression in the ACC, which was upregulated from day 3 post‐surgery and peaked on day 7 (Figure 1C). This spatiotemporally specific expression pattern indicates the potential role of GPR35 in NP chronicity.

A systematic investigation using gain‐ and loss‐of‐function experimental paradigms has established that GPR35 exerts bidirectional regulatory effects on NP: GPR35 knockdown exacerbated pain hypersensitivity and depression‐like behaviors, while overexpression significantly alleviated these effects (Figures 2 and 3). These findings are consistent with those of previous studies showing the involvement of GPR35 in pain modulation through the regulation of neuronal excitability and neurotransmitter release [29, 30]. Our study of the connection between the function of GPR35 and the co‐occurring emotional conditions in patients with NP has expanded our understanding of its pathological physiological role.

Transcriptome sequencing and molecular validation experiments demonstrated that GPR35 exerted neuroprotective effects through the Nr4a1/PI3K/AKT signaling axis (Figures 4 and 5). Notably, GPR35 activates the upregulation of Nr4a1 expression, thereby suppressing neuroinflammation and alleviating depression‐like behaviors. This discovery provides a novel molecular basis for understanding the mechanism underlying the “pain‐depression comorbidity” in NP.

L‐Kynurenine (L‐Kyna), an endogenous GPR35 agonist, exhibits blood–brain barrier permeability and mediates pleiotropic neurobiological effects within the central nervous system [31, 32]. A pivotal discovery of this investigation was the therapeutic efficacy of L‐Kyna; it significantly attenuated both nociceptive hypersensitivity (PWT and PWL) and affective dysregulation in CCI mice (Figure 7). Those findings provide direct evidence for the development of GPR35‐based therapeutic strategies for NP.

In recent years, GPR35 has gained significant attention as a novel molecular target for NP treatment. Although substantial progress has been made in understanding its ligand recognition and signal transduction mechanisms, the mechanisms of GPR35 in NP remain to be clarified. Notably, GPR35 exhibits a multi‐cellular expression profile in the central nervous system and is localized in neurons, astrocytes, and microglia [33, 34, 35]. This study revealed that CCI‐induced changes in GPR35 expression involve neuroglial‐neuronal interactions (Figure S1), suggesting cell‐type specificity in its regulatory network.

Further studies revealed that the conditional modulation of GPR35 expression in the ACC bidirectionally influences NP phenotypes. GPR35 knockdown induced microglial overactivation (increased CD68^+^ cells), a pro‐inflammatory cytokine storm, ultimately exacerbating the pain‐depression comorbidity (Figures 2 and 3). This discovery provides critical insights into the central regulatory network of GPR35, and its functional implementation may rely on the activation of the Nr4a1/PI3K/AKT signaling axis, regulation of microglial phenotypic switching, and neuroinflammatory homeostasis mediated by glial‐neuronal interactions.

The analgesic mechanism of GPR35 involves a multi‐level neural regulatory network. At the molecular level, GPR35 activation inhibits neuronal excitability and pain signal transmission [36]. At the neural circuit level, GPR35 expression in peripheral nerves suggests a potential peripheral analgesic role by modulating the excitability of primary afferent neurons. Furthermore, GPR35 expression in the central pain modulatory systems (e.g., the PAG‐RVM pathway) indicates its capacity to regulate spinal nociceptive processing by modulating GABAergic interneuron activity and decreasing 5‐HTergic inhibitory system functions [21]. Moreover, GPR35 participates in gut‐brain axis metabolic regulation by affecting microbial metabolite signaling to modulate neuronal plasticity and emotional behaviors [35]. This discovery expands the functional scope of GPR35 from nociception regulation to broad‐spectrum therapeutic targets in neuropsychiatric disorders. Although GPR35 cannot completely block the progression of pain and depression, the prophylactic therapeutic potential of its agonist, L‐Kyna, offers a novel strategy for early intervention in NP.

In this study, GPR35 knockout induced significant transcriptional changes in the ACC, with the Nr4a1/PI3K/AKT signaling axis identified as the key downstream pathway (Figures 4 and 5). Additionally, GPR35 suppressed pro‐inflammatory cytokine release in the ACC and enhanced neuronal synaptic plasticity through the positive regulation of Nr4a1 expression, thereby synergistically alleviating pain hypersensitivity and depression‐like behaviors (Figure 6). This discovery fills a critical gap in the understanding of GPCR regulatory networks in the ACC in NP research.

In this study, Nr4a1, a member of the nuclear receptor superfamily, was identified as a critical molecular hub that mediates GPR35‐dependent neuroinflammatory regulation. Nr4a1 exerts anti‐inflammatory and neuroprotective effects under various pathological conditions [37, 38]; however, its regulatory mechanisms in NP remained unclear. We provide the evidence of expression dynamics regarding CCI‐induced Nr4a1 expression changes in the ACC and pain‐depression comorbidity progression in NP (Figure 5D,E), and that Nr4a1 knockdown abolished GPR35‐mediated analgesic and antidepressant effects (Figure 6). These findings revealed that Nr4a1 is an NP‐specific therapeutic target, rather than a broad‐spectrum anti‐inflammatory factor. Integrating reported roles of Nr4a1 in pain signaling [39, 40, 41], we propose the “GPR35‐Nr4a1 inflammation‐plasticity regulatory axis” hypothesis. This axis disrupts the vicious pain‐depression cycle via dual mechanisms (neuroinflammatory suppression and maintenance of synaptic homeostasis).

GPR35 co‐localizes with TRPA1 in the peripheral nervous system and exerts analgesic effects by modulating visceral mechanoreceptor activity [33]. This finding suggests GPR35 may have tissue‐specific functions: primarily regulating visceral pain in the periphery, while potentially mediating NP chronicity through the ACC in the central nervous system. Notably, GPR35 agonists exhibit unique therapeutic advantages by alleviating chronic pain‐related nociceptor sensitization without affecting acute pain transmission, making them ideal targets for chronic pain treatment. In this study, L‐Kyna activates ACC GPR35 to significantly increase mechanical pain thresholds and thermal pain latencies, providing neuroprotection and suppressing neuroinflammation. These experimental findings provide robust preclinical evidence supporting the therapeutic potential and clinical translatability of GPR35‐targeted pharmacological interventions.

Despite the findings, this study has the following limitations: (1) the heat pain threshold test relies on operator observation, which may introduce subjective bias; (2) the mouse models cannot fully simulate the complexity of chronic pain in humans; and (3) the small sample size may lead to statistical errors. Future studies should incorporate multispecies models and objective monitoring techniques to improve the reliability of the results.

## 5 Conclusion

Our findings demonstrated that GPR35 acts as a protective factor against NP and pain‐related affective disturbances in the ACC by modulating the activation of the downstream Nr4a1 target gene, which activates the PI3K/AKT pathway and suppresses neuroinflammation. Additionally, activation of GPR35 using L‐Kyna provides analgesic and antidepressant benefits. Overall, targeting the GPR35/Nr4a1/PI3K/AKT regulatory axis may be a novel approach for improving the prognosis of NP.

## Author Contributions

Bin Wang, Weidong Yao, and Yongquan Chen designed and supervised the study and critically revised the manuscript. Jianling Xu and Jingyong Zhou performed the experiments, collected and analyzed the data, and drafted the manuscript. Xiaojun Li contributed to the experimental design and revised the manuscript. Tingting Qu and Changjian Zheng collected the clinical data and revised the manuscript. Qingyu Cheng and Xiuyang Lei performed the animal experiments and collected the data. All authors read and approved the final manuscript.

## Ethics Statement

This study strictly adhered to the guidelines of the International Association for the Study of Pain and Animal Research: Reporting of In Vivo Experiments guidelines. It is registered in the Chinese Clinical Trial Registry (approval number: ChiCTR2500113757). All animal experimental protocols were reviewed and approved by the Experimental Animal Welfare and Ethics Committee of Wannan Medical College (approval number: WNMC‐AWE‐2023417) and the Medical Ethics Committee (approval number: 2023‐176).

## Consent

Each human participant signed the informed consent form.

## Conflicts of Interest

The authors declare no conflicts of interest.

## Supporting information


**Data S1:** cns70852‐sup‐0001‐Supinfo.doc.

## Data Availability

All data are available upon reasonable request.
