# Supplementary material for: Activation of GPR35 in the Anterior Cingulate Cortex Alleviates Neuropathic Pain and Depression‐Related Behavior
Source: CNS Neurosci Ther. 2026 Mar 31;32(4):e70852. doi: 10.1002/cns.70852 (PMC13140911; doi:10.1002/cns.70852)
Supplement: Supplementary file 1 — Data S1: cns70852‐sup‐0001‐Supinfo.doc. [file CNS-32-e70852-s001.doc]

**Supporting information**

**Activation of GPR35 in the Anterior Cingulate Cortex Alleviates Neuropathic Pain and Depression-Related Behavior**

Jianling Xu^1#^, Jingyong Zhou^1,2#^, Xiaojun Li^1^, Tingting Qu^3^, Changjian Zheng^1,2^, Qingyu Cheng^1^, Xiuyang Lei^1^, Weidong Yao^1^*, Yongquan Chen^1,2^*, Bin Wang^1,2,4^*

^1^Department of Anesthesiology, The First Affiliated Hospital of Wannan Medical College, Yijishan Hospital, Wuhu, China

^2^Department of Pain Medicine, The First Affiliated Hospital of Wannan Medical College, Yijishan Hospital, Wuhu, China

^3^Department of Neurology, The First Affiliated Hospital of Anhui Medical University, Hefei, China

^4^Anhui Province Key Laboratory of Non-coding RNA Basic and Clinical Transformation, Wuhu, China

**Materials and** **Methods**

**Co-immunoprecipitation**

The brain tissue was lysed in RIPA buffer (containing protease/phosphatase inhibitors). The lysate was incubated with magnetic beads that bound to the target antibody overnight at 4 °C, followed by washing to remove non-specific binding substances. The proteins from co-immunoprecipitation were eluted and separated by SDS-PAGE, and then detected by western blotting. The magnetic beads bound to IgG served as a negative control. The protein interactions were verified by reverse co-immunoprecipitation and band intensity quantification. The experiment was repeated three times, and the results were consistent, ensuring specificity and reproducibility.

**RNA-seq**

Total RNA was extracted from the ACC samples of mice cryopreserved in liquid nitrogen (stored at -80 °C) using TRIzol reagent. RNA quality was verified by Nanodrop (OD260/OD280: 1.8–2.0) and Agilent 2100 Bioanalyzer (RIN ≥ 7.0). mRNA enrichment, fragmentation, and cDNA synthesis were followed by library construction (end repair, A-tailing, adapter ligation, PCR amplification) with 150–300 bp fragment selection. Paired-end 150 bp sequencing was performed on Illumina NovaSeq 6000 ( ≥ 6 G/sample). Clean reads were aligned to GRCh38 via STAR, and gene expression was quantified by featureCounts (FPKM). DEGs were identified using DESeq2 (|log2FC| ≥ 1, FDR < 0.05) and subjected to GO/KEGG enrichment. Three biological replicates (PCA-validated reproducibility) and qRT-PCR (r ≥ 0.8) confirmed results.

**Molecular docking**

Molecular Docking Assay GPR35 crystal structure was retrieved from the PDB database (PDB ID: 6OIN, resolution: 2.8 Å), and Nr4a1 structure was constructed via SWISS-MODEL using PDB ID: 3F1P (Nr4a2, resolution: 2.3 Å) as the template (sequence identity: 78%, coverage: 85%). Sequence alignment (Clustal X 2.1) showed conserved DNA-binding and ligand-binding domains between Nr4a1 and the template. Both structures were processed with PyMOL 1.8 (remove water molecules, ligands, and redundant chains) and AutoDockTools 1.5.6 (add polar hydrogens and Gasteiger charges). The GPR35 active pocket was defined based on literature (residues Ala102–Phe110, Asn254–Ser262), with a grid box of 40 × 40 × 40 Å (center coordinates: X = 15.2, Y = 28.7, Z = 32.1). Molecular docking was performed using AutoDock Vina 1.2.0 (exhaustiveness = 16, num_modes = 10). Top 5 conformations with binding energy ≤ -6.5 kcal/mol were selected. Hydrogen bonds and hydrophobic interactions were analyzed by LigPlot + 4.5.3, and conformational stability was verified by RMSD < 1.5 Å. Experiments were repeated 3 times for reliability.

**1. Supporting tables**

Table S1 List primers for q-PCR.

| Gene(mouse) | Forward primer (5′–3′) | Reverse primer (5′–3′) |
| --- | --- | --- |
| GPR35 | CAGTGCCAGTGCCGTCCTAATG | GTGGCTGGCTTGGACGCTTC |
| Nr4a1 | GAAAGTTGGGGGAGTGTGCT | GGGTCTCATCTAATGGGCCG |
| GAPDH | TGTGTCCGTCGTGGATCTGA | TTGCTGTTGAAGTCGCAGGAG |

Table S2 Demographic clinical characteristics of supplement subjects (*n* = 20)

|  | NP (*n* = 20) | Con (*n* = 20) | *P*-value |
| --- | --- | --- | --- |
| Sex, male, n (%) | 11（0.55） | 13（65.0） | 0.53 |
| Age, y | 48.4±13.0 | 51.6±9.1 | 0.36 |
| Height | 165.8±7.19 | 166.6±7.1 | 0.71 |
| Weight | 63.3±7.78 | 66.2±10.8 | 0.35 |
| BMI | 23.0±2.4 | 23.8±3.3 | 0.40 |

Note: There was no significant difference in demographic characteristics between the patients with neuropathic pain and the control group


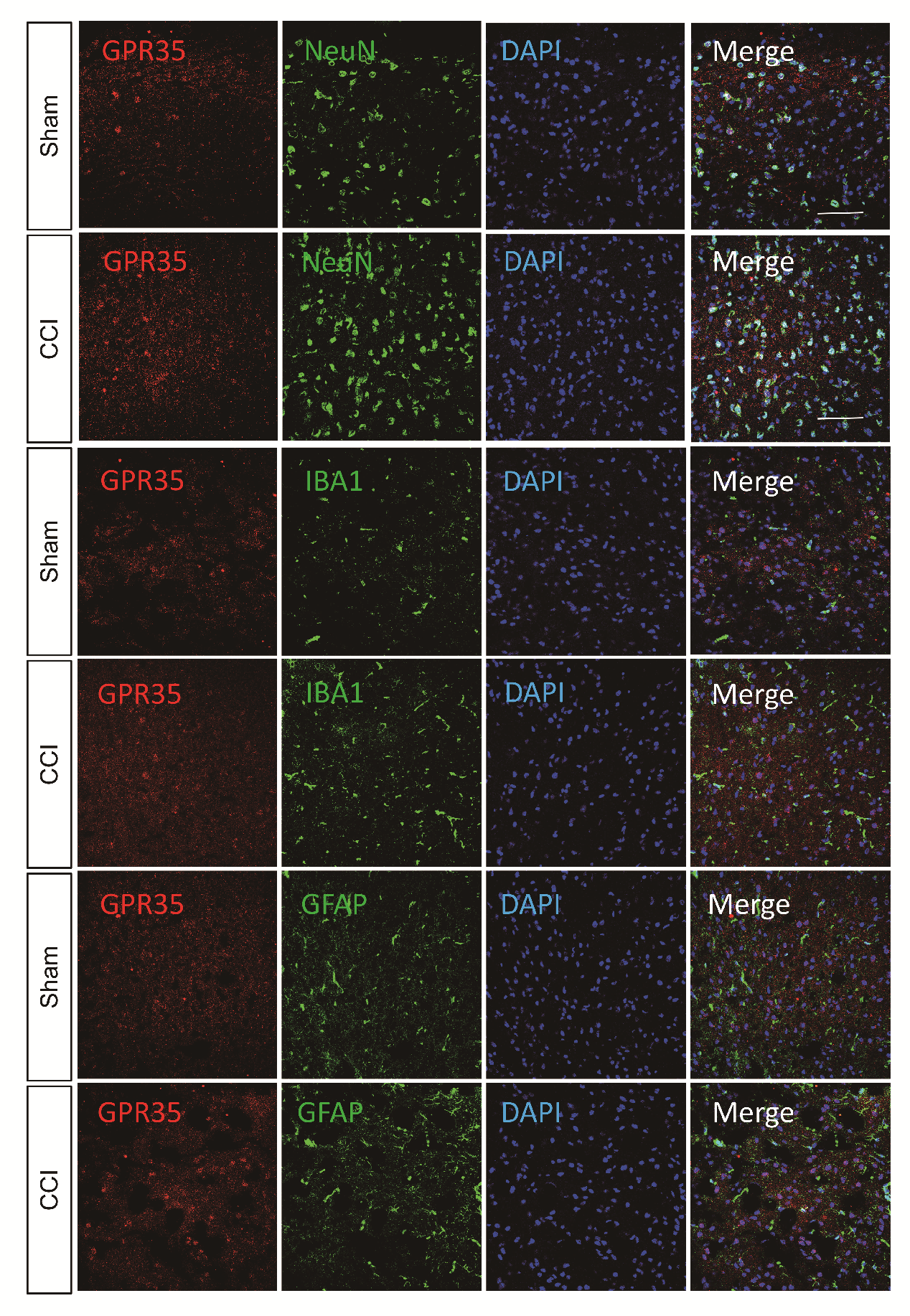


FIGURE S1. GPR35 is expressed in neurons, microglia and astrocytes. GPR35 is mainly co-localized with neurons and microglia; after sham operation and chronic traumatic brain injury (CCI), GPR35 co-localizes with NeuN, Iba1 and GFAP. Representative images of immunofluorescence staining show the expression of GPR35 (red), NeuN (green), Iba1 (green) and GFAP (green), including their overlapping areas, represented by rectangles. On the 7th day after chronic traumatic brain injury (CCI), brain tissue samples were collected from the right anterior cingulate cortex region. scale bar: 20 μm. *n* = 4.


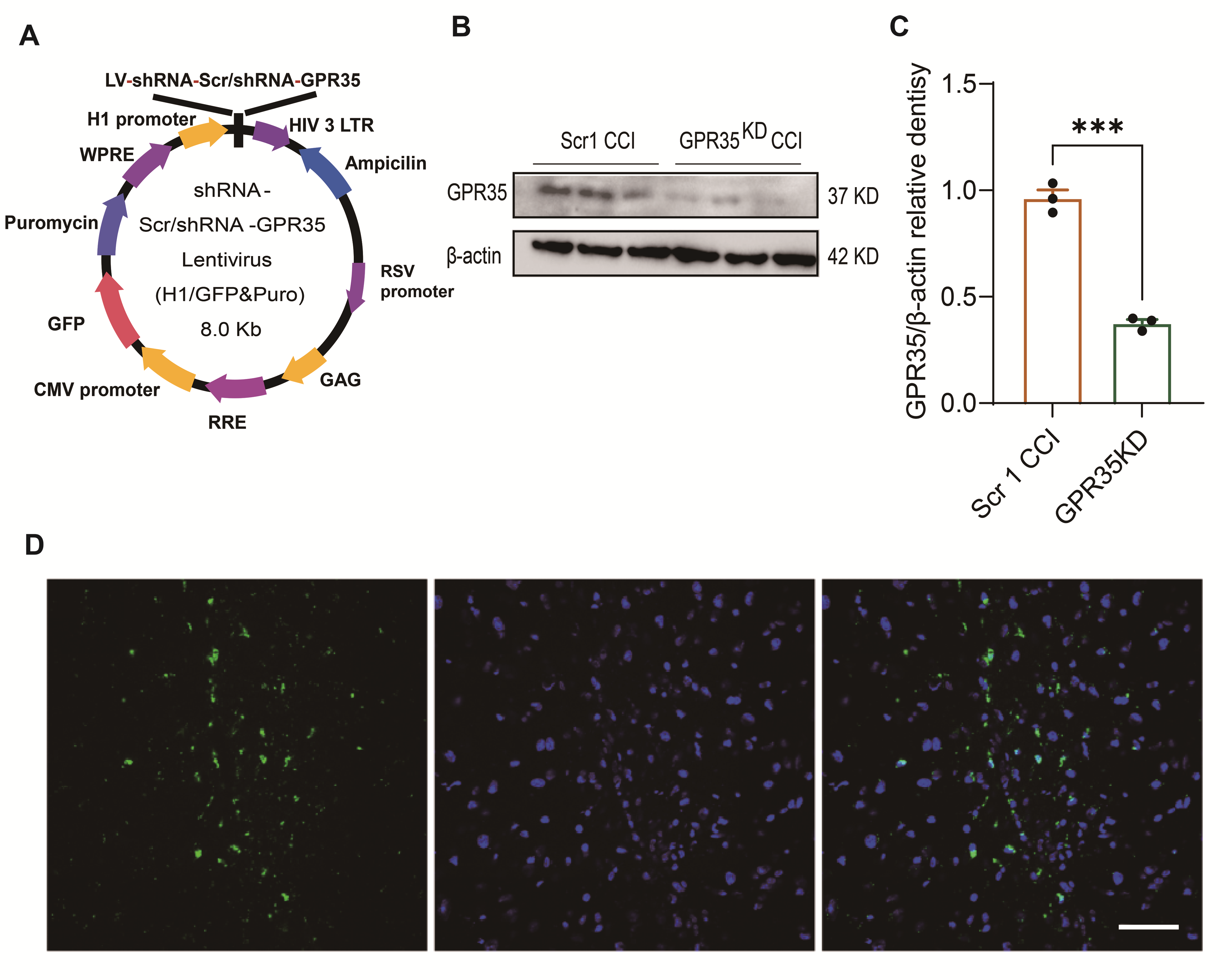


FIGURE S2. Validation of the knockdown effect of GPR35. (A) Schematic diagram of GPR35 knockdown virus. (B,C) Seven days after the injection of GPR35 knockdown virus, the protein expression was significantly reduced. *n =* 3, ****P <* 0.001. (D) The expression of GPR35 in the ACC region after viral injection. *n* = 4.


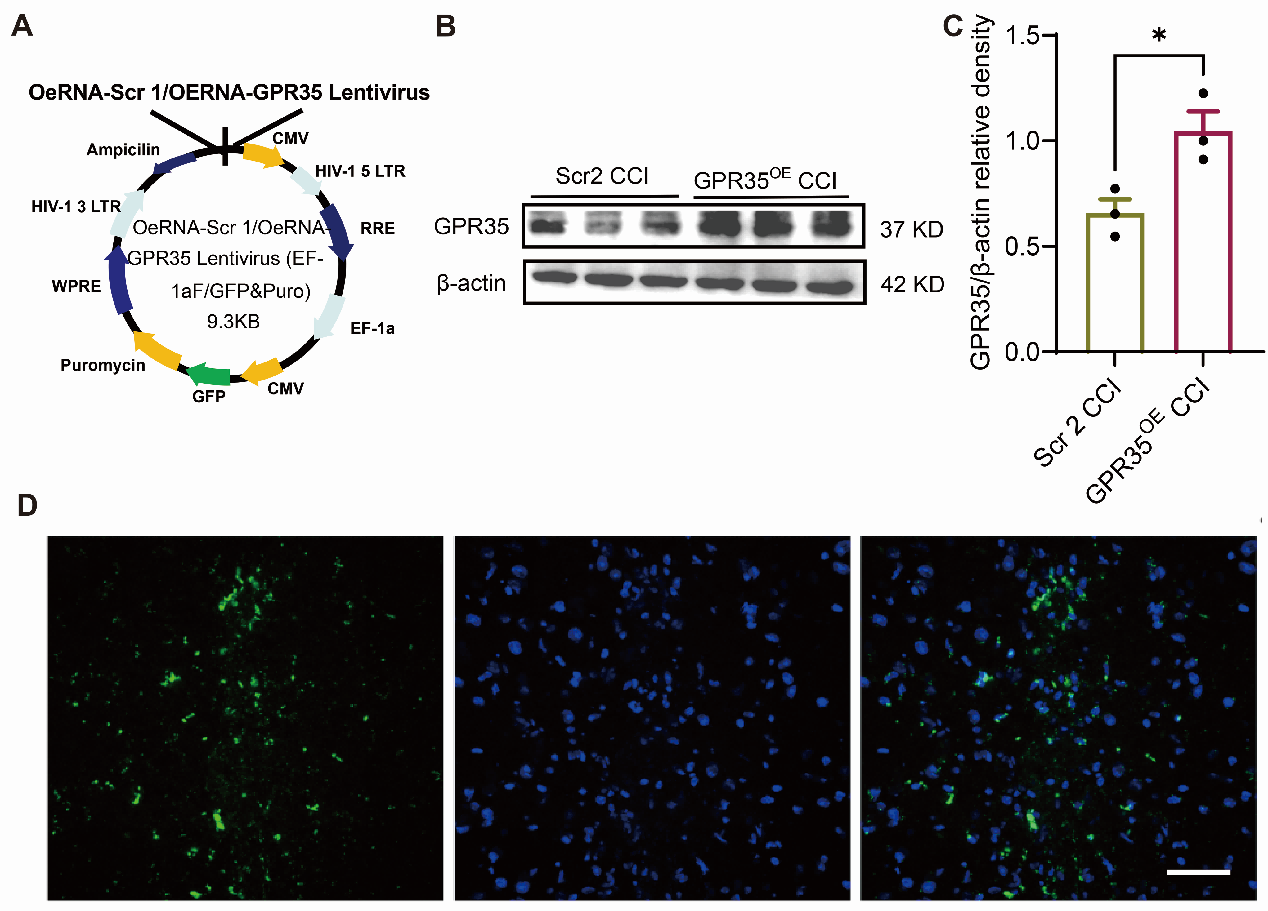
FIGURE S3. Verification of the overexpression effect of GPR35. (A) Schematic diagram of GPR35 overexpression virus. (B,C) Seven days after the injection of GPR35 overexpression virus, the protein expression was significantly increase. *n =* 3, **P <* 0.05. (D) The expression of GPR35 in the ACC region after viral injection. *n* = 4.


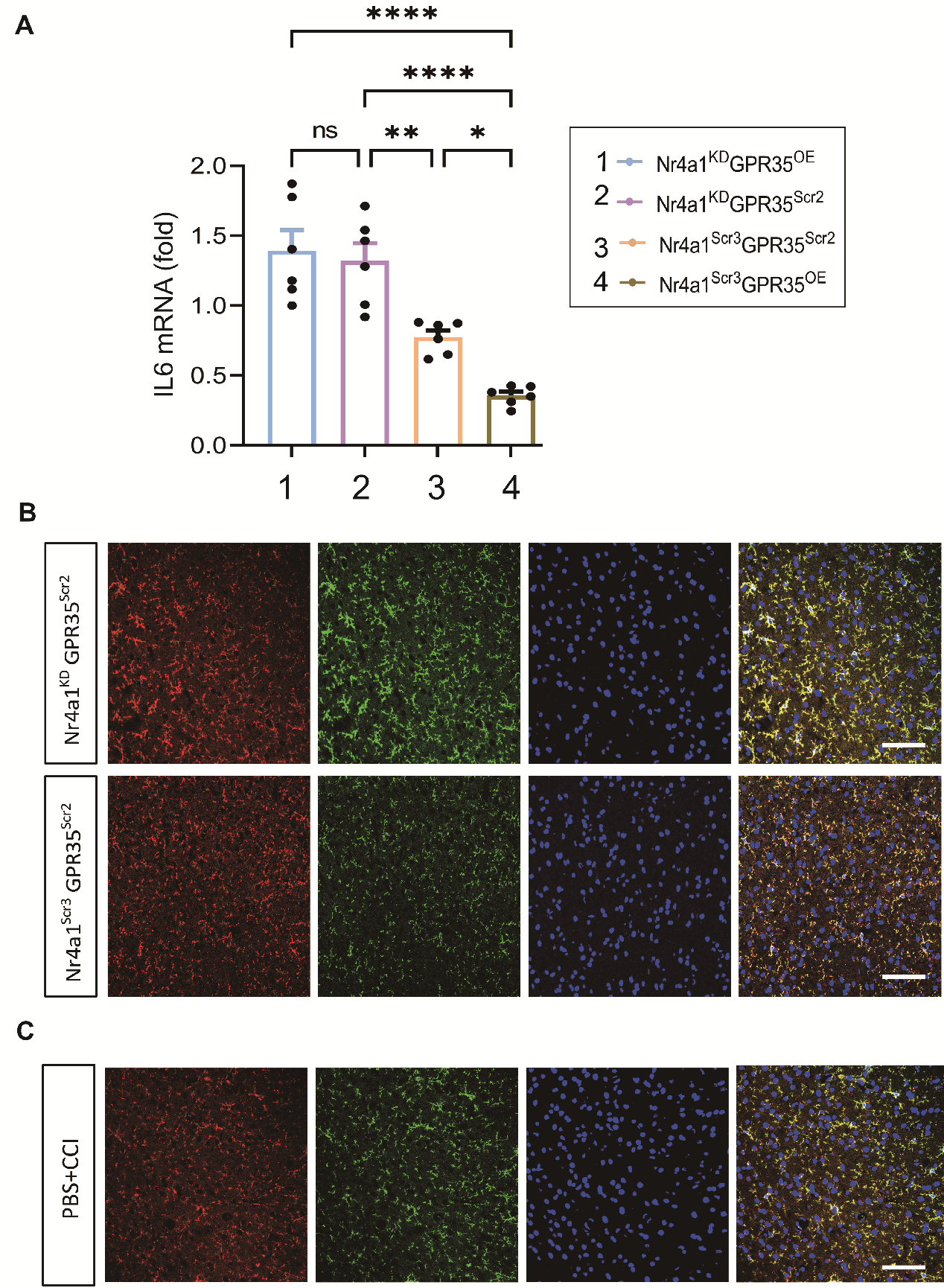


FIGURE S4. (A) Knocking down the Nr4a1 gene partially counteracted the protective effect of GPR35 gene overexpression in reducing IL6 levels. **P <* 0.05, ***P <* 0.01, *****P <* 0.001. (B) When Nr4a1 was knocked down, the activation level of microglia increased, which was confirmed by the increased co-staining of CD68^+^ and IBA1^+^. *P* < 0.05. (C) In the ACC region, PBS-treated CCI mice showed CD68^+^/IBA1^+^ double-positive microglia. Scale bar: 20 μm.


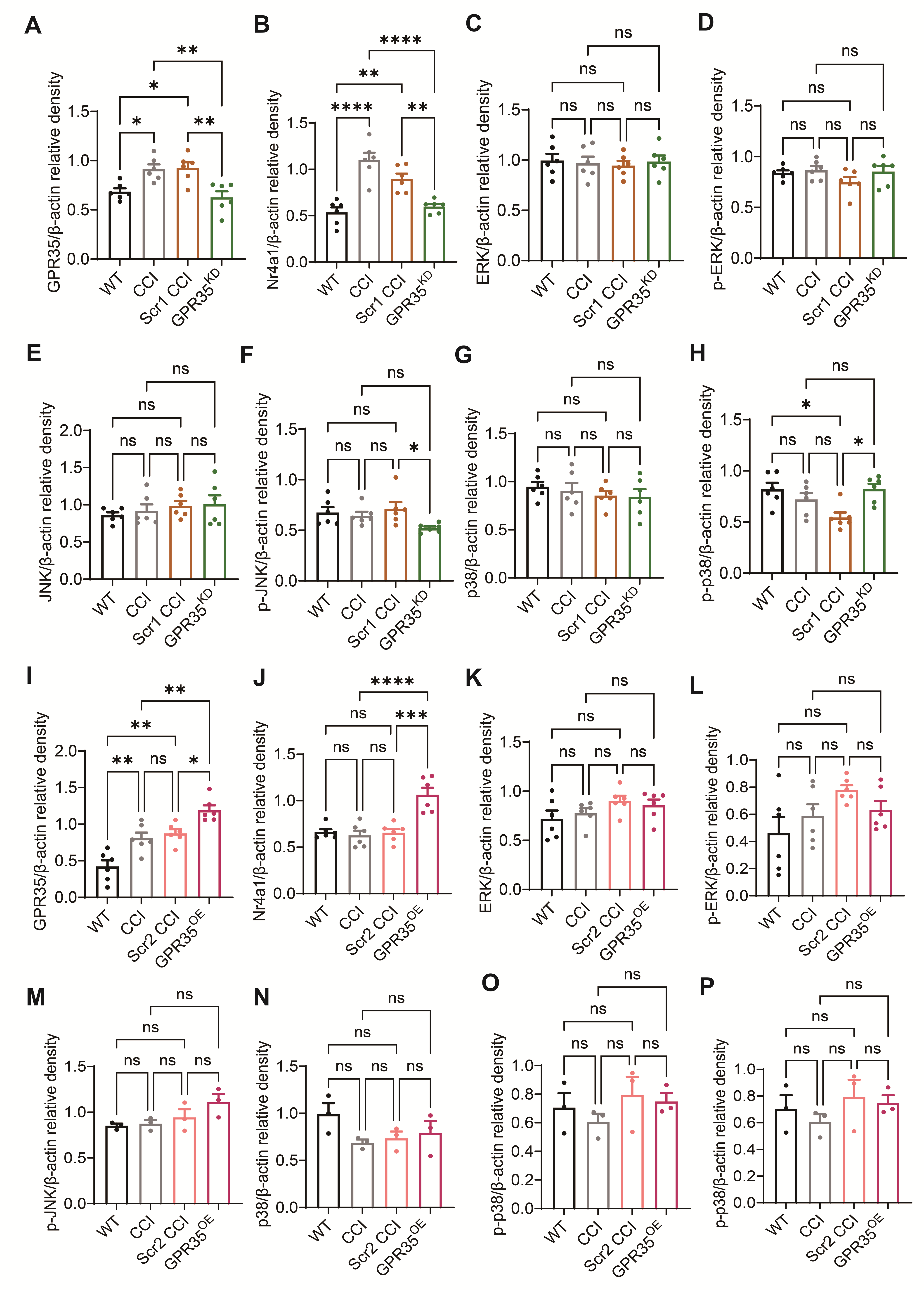


FIGURE S5. GPR35 regulates pain and depression-like behaviors in mice through the Nr4a1/PI3K/AKT pathway. (A-P) The changes in protein expression after knockdown or overexpression of GPR35.
